# Association of an Alphasatellite with *Tomato* *Yellow* *Leaf* *Curl* *Virus* and *Ageratum* *Yellow* *Vein* *Virus* in Japan is Suggestive of a Recent Introduction

**DOI:** 10.3390/v6010189

**Published:** 2014-01-14

**Authors:** Muhammad Shafiq Shahid, Masato Ikegami, Abdul Waheed, Rob W. Briddon, Keiko T. Natsuaki

**Affiliations:** 1 Department of International Agricultural Development, Tokyo University of Agriculture, Tokyo 156-8502, Japan; E-Mails: mikegam@77.catv-yokohama.ne.jp (M.I.); keiko@nodai.ac.jp (K.T.N.); 2 Department of Biosciences, COMSATS Institute of Information Technology, Sahiwal 57000, Pakistan; E-Mail: director@ciitsahiwal.edu.pk; 3 Agricultural Biotechnology Division, National Institute for Biotechnology and Genetic Engineering, Faisalabad 38000, Pakistan; E-Mail: rob.briddon@nibge.org

**Keywords:** geminivirus, begomovirus, betasatellite, alphasatellite, tomato

## Abstract

Samples were collected in 2011 from tomato plants exhibiting typical tomato leaf curl disease symptoms in the vicinity of Komae, Japan. PCR mediated amplification, cloning and sequencing of all begomovirus components from two plants from different fields showed the plants to be infected by *Tomato* *yellow* *leaf* *curl* *virus* (TYLCV) and *Ageratum* *yellow* *vein* *virus* (AYVV). Both viruses have previously been shown to be present in Japan, although this is the first identification of AYVV on mainland Japan; the virus previously having been shown to be present on the Okinawa Islands. The plant harboring AYVV was also shown to contain the betasatellite Tomato leaf curl Java betasatellite (ToLCJaB), a satellite not previously shown to be present in Japan. No betasatellite was associated with the TYLCV infected tomato plants analyzed here, consistent with earlier findings for this virus in Japan. Surprisingly both plants were also found to harbor an alphasatellite; no alphasatellites having previously been reported from Japan. The alphasatellite associated with both viruses was shown to be Sida yellow vein China alphasatellite which has previously only been identified in the Yunnan Province of China and Nepal. The results suggest that further begomoviruses, and their associated satellites, are being introduced to Japan. The significance of these findings is discussed.

## 1. Introduction

The family *Geminiviridae* consists of phytopathogenic viruses with circular single-stranded (ss) DNA genomes that are encapsidated in characteristic twinned quasi-icosahedral particles. At this time, the family encompasses seven genera (*Becurtovirus*, *Eragrovirus*, *Turncurtovirus*, *Topocuvirus*, *Curtovirus*, *Mastrevirus* and *Begomovirus*), with viruses assigned based upon genome arrangement, insect vector and sequence relatedness [1,2]. Geminiviruses of the genus *Begomovirus*, transmitted exclusively by whitefly *Bemisia* *tabaci*, are the most numerous and economically the most damaging. Most begomoviruses originating from the New World (NW) have genomes consisting of two components, known as DNA A and DNA B, both of which are required for virus infectivity, although recently a monopartite begomovirus (with a genome consisting of only a homolog of the DNA A components of bipartite viruses) has been identified [3,4]. In the Old World (OW), however, although a small number of bipartite begomoviruses have been identified, the majority are monopartite. A small proportion of these monopartite begomoviruses are truly monopartite; their single component genomes inducing disease in plants in the field, such as *Tomato* *leaf* *curl* *virus* in Australia [5]. The majority of monopartite OW begomoviruses associate with a class of ssDNA satellites known as betasatellites (earlier known as DNA β) [6].

Betasatellites are small (~1,350 nt) ssDNA satellites that have only been identified in the OW [7]. They are dependent on the helper begomovirus for their replication, movement in plants and transmission by the whitefly vector [6]. They encode a single gene in the complementary-sense (known as βC1), contain a region of sequence rich in adenine (A-rich) and an approximately 100–150 nucleotide sequence (known as the satellite conserved region [SCR]) conserved between all betasatellites. The SCR encompasses a predicted hairpin structure containing, within the loop, the motif TAATATTAC (known as the nonanucleotide sequence), with similarity to the origin of virion-strand DNA replication of the geminiviruses [8]. The betasatellites are very diverse and all functions, including suppression of post-transcriptional gene silencing and possible virus movement, have been shown to be mediated by βC1 [9,10].

In addition to betasatellites, many begomovirus-betasatellite complexes are also associated with a third ssDNA component for which the collective term alphasatellite has been proposed (earlier known as DNA 1). Alphasatellites are satellite-like, circular ssDNA molecules ~1,400 nt in length. They have a single gene, encoding the replication-associated protein (Rep; a rolling-circle replication initiator protein), and are capable of autonomous replication in plant cells. Closely related to the Rep-encoding components of nanoviruses (a second family of plant infecting ssDNA viruses [11]), from which they are believed to have evolved, they require a helper begomovirus for movement within and between plants [12,13]. Recently, two distinct alphasatellites have been identified in association with bipartite begomoviruses in the NW [14,15].

A number of monopartite begomoviruses have been identified in Japan. These include *Tobacco* *leaf* *curl* *Japan* *virus* (TbLCJV) and *Honeysuckle* *yellow* *vein* *mosaic* *virus* (HYVMV), which cause tomato yellow dwarf disease [16,17], and *Ageratum* *yellow* *vein* *virus* (AYVV), which has so far only been reported from Okinawa and causes problems in tomato there [18]. The most agriculturally significant begomovirus present in Japan is *Tomato* *yellow* *leaf* *curl* *virus* (TYLCV); a virus that has its origins in the Middle East/Mediterranean region and has spread globally, including to Japan, and was first reported in Japan in 1998 [19,20]. Although TbLCJV, HYVMV and AYVV are associated with betasatellites [17,18], TYLCV is generally not associated with satellites although a single isolate, originating from Oman, has been shown to be associated with both an alpha- and a betasatellite [21]. Interestingly, to date, no alphasatellites have been reported from Japan. The study presented here shows the presence of an alphasatellite in tomato in the presence of TYLCV.

## 2. Results and Discussion

### 2.1. Results

#### 2.1.1. Cloning, Sequencing and Sequence Analysis of Begomoviruses

**Figure 1:**
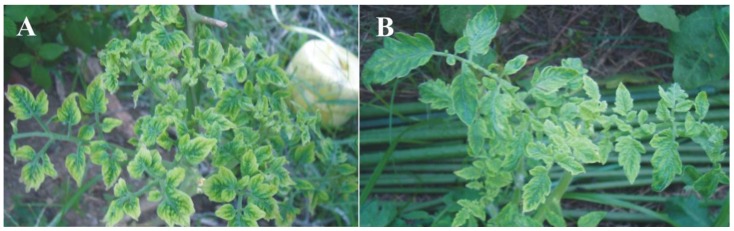
Symptoms exhibited by field-collected tomato plants originating from Komae, Japan. (**A**) Plants infected with *Tomato* *yellow* *leaf* *curl* *virus* (TYLCV) and Sida yellow vein China alphasatellite (SiYVCNA); and (**B**) plants infected with *Ageratum* *yellow* *vein* *virus* (AYVV), Tomato leaf curl Java betasatellite (ToLCJaB) and SiYVCNA.

**Figure 2:**
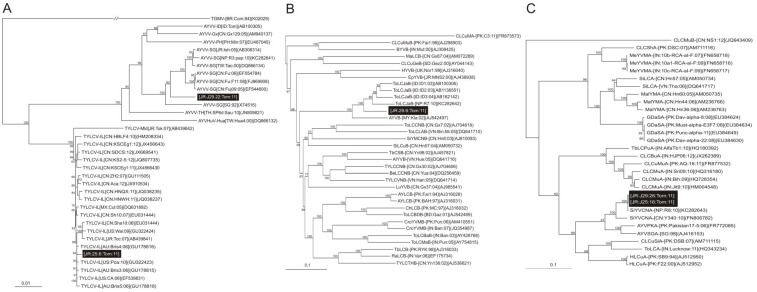
Phylogenetic dendrograms based upon alignments of selected (**A**) begomovirus genome (or DNA A component) sequences; (**B**) betasatellite sequences; and (**C**) alphasatellite sequences with those identified here.

In 2011, tomato plants with symptoms typical of begomoviruses, consisting of vein yellowing, foliar chlorosis, leaf curling and stunting, were observed in Komae, Tokyo, Japan (Figure 1). Four plants from two distinct locations (A: Central Komae and B: South West of Komae) were collected and diagnostic PCR indicated the presence of begomoviruses. PCR amplification with specific primers (TYLCVF/TYLCVR) from plant A yielded a single product of the expected size (~2.8 kb) which was cloned into the pGEM-T vector. Three clones were obtained which all showed identical restriction patterns and partial sequencing revealed them to have near identical sequences (results not shown). For this reason one clone (J25.6) was selected for complete sequencing. The complete sequence was determined to be 2,784 nt and is available in the databases under the accession number KC677732. Sequence comparisons showed the sequence to be closely related to isolates of TYLCV (79.1 to 99.4% nucleotide sequence identity to all TYLCV sequences available in the databases) with the highest to a TYLCV isolate originating from Australia (GU178816). This indicates that the virus from tomato is an isolate of TYLCV for which we propose the isolate descriptor [Japan:Komae:2011] (TYLCV-[JR:Kom:11]).The sequence has a genome organization typical of monopartite begomoviruses (or DNA A components of bipartite begomoviruses) originating from the OW with two genes encoded in the virion-sense, the coat protein (CP) and V2 protein, and four genes encoded in the complementary-sense, Rep, the transcriptional activator protein (TrAP), the replication enhancer protein (REn) and the C4 protein (Table 1). Between the virion- and complementary-sense genes lies a non-coding, intergenic region (IR) that contains a predicted stem-loop structure with the nonanucleotide sequence “TAATATTAC” forming part of the loop. This motif forms part of the origin of virion-strand DNA replication [22]. A phylogenetic tree, based on an alignment of the complete nucleotide sequence of J25.6 with selected genomes (or DNA A components) of begomoviruses from the databases, shows the virus from tomato to segregate with the isolates of TYLCV included in the analysis and to be distinct from other begomoviruses, confirming its identification as an isolate TYLCV (Figure 2A).

**Table 1:** Features of the begomovirus clones obtained in this study.

Isolate	Virus Species	Size (nt)	Genes (coordinates/coding capacity [no. of amino acids]/predicted size of product [kDa])					
			CP	V2	Rep	TrAP	REn	C4
J29.22	AYVV	2,751	291-1064	131-481/	2601-1513	1613-1206	1465-1061	2444-2154
			/257/29.9	116/13.5	/362/40.8	/135/15.5	/134/16.1	/96/10.8
J25.6	TYLCV	2,784	308-1084	148-498	2618-1542	1633-1226	1485-1081	2467-2171
			/258/30.1	/116/13.5	/358/41.0	/135/15.6	/134/15.9	/98/11.2

The complete nucleotide sequence of the begomovirus (isolate J29.22) isolated from tomato plant B was determined to be 2,751 nt in length (KC677733). Sequence comparisons showed the sequence to have the highest levels of identity (79.6 to 96.8) to isolates of AYVV viruses available in the databases (46 available at this time) with the highest (96.8%) to an isolate also originating from tomato from Japan (AB306314) [18]. The virus cloned from tomato has a genome organization typical of OW monopartite begomoviruses (or DNA A components of bipartite begomoviruses; Table 1). The phylogenetic analysis (Figure 2A) shows the begomovirus isolated from tomato to segregate with isolates of AYVV, confirming it identification as an isolate of this species. The isolate descriptor [Japan:Komae:tomato:2011] ([JR:Kom:tom:11]) is proposed for this sequence.

Vertical branches are arbitrary, horizontal branches are proportional to calculated mutation distance. Values at nodes indicate percentage bootstrap values (1,000 replicates). The dendrograms were arbitrarily rooted on the sequences of isolates of *Tomato* *golden* *mosaic* *virus* (TGMV), Cotton leaf curl Multan alphasatellite (CLCuMA) or Cotton leaf curl Multan betasatellite (CLCuMuB) as outgroups, respectively. The sequences produced as part of the study presented here are highlighted by black boxes. The begomovirus acronyms used are *Ageratum* *yellow* *vein* *virus* (AYVV), *Ageratum* *yellow* *vein* *China* *virus* (AYVCNV), *Ageratum* *yellow* *vein* *Hulian* *virus* (AYVHuV), and *Tomato* *yellow* *leaf* *curl* *virus* (TYLCV). The betasatellite acronyms used are Ageratum yellow vein betasatellite (AYVB), Alternanthera yellow vein betasatellite (AlYVB), Ageratum yellow leaf curl betasatellite (AYLCB), Bean leaf curl China betasatellite (BeLCCNB), Bhendi yellow vein betasatellite BYVB, Chili leaf curl betasatellite (ChLCB), Cotton leaf curl Gezira betasatellite (CLCuGeB), Croton yellow vein mosaic betasatellite (CroYVMB), Eupatorium yellow vein betasatellite EpYVB, Honeysuckle yellow vein betasatellite HYVB, Ludwigia yellow vein betasatellite (LuYVB), Malvastrum leaf curl betasatellite (MaLCB), Radish leaf curl betasatellite (RaLCB), Sida leaf curl betasatellite (SiLCuB), Sida yellow mosaic China betasatellite (SYMCNB), Tobacco curly shoot betasatellite (TbCSB), Tobacco leaf curl betasatellite (TbLCB), Tomato leaf curl Bangalore betasatellite (ToLCBaB), Tomato leaf curl leaf curl Bangladesh betasatellite (ToLCBDB), Tomato leaf curl China betasatellite (ToLCCNB), Tomato leaf curl Java betasatellite (ToLCJaB), Tomato leaf curl Laos betasatellite (ToLCLAB), Tomato leaf curl Maharastra betasatellite (ToLCMaB), Tomato yellow leaf curl China betasatellite (TYLCCNB), Tomato yellow leaf curl Thailand betasatellite (TYLCTHB), Tomato yellow leaf curl Vietnam betasatellite (TYLCVNB). The alphasatellite acronyms used are Ageratum yellow vein Kenya alphasatellite (AYVKA), Ageratum yellow vein Singapore alphasatellite (AYVSGA), Cotton leaf curl Dabwali alphasatellite (CLCuDaA), Cotton leaf curl Gezira alphasatellite (CLCuGeA), Cotton leaf curl Multan alphasatellite (CLCuMA), Cotton leaf curl Shahdadpur alphasatellite (CLCuShA), Duranta leaf curl alphasatellite (DuLCA), Gossypium darwinii symptomless alphasatellite (GDarSLA), Hibiscus leaf curl alphasatellite (HLCuA), Malvastrum yellow mosaic alphasatellite (MaYMA), Okra leaf curl alphasatellite (OLCuA), Okra leaf curl Mali alphasatellite (OLCuMA), Okra leaf curl India alphasatellite (OLCuIA), Sida yellow vein alphasatellite (SiYVA), Sida yellow vein Vietnam alphasatellite (SiYVVA), Tomato leaf curl alphasatellite (ToLCA), Tomato yellow leaf curl China alphasatellite (TYLCCNA) and Tomato yellow leaf curl Yunnan alphasatellite (TYLCYnA). In each case the database accession number and isolate descriptor is given.

#### 2.1.2. Analysis of Betasatellite

The betasatellite (isolate J29.6) isolated from tomato infected with AYVV (plant B) was determined to be 1,363 nt in length (KC677734). The sequence contained all the features typical of betasatellites; a single conserved gene (known as βC1; coordinates 546-190) in the complementary-sense with the capacity to encode 118 amino acid protein, a region of sequence rich in adenine (722 to 1016) and a sequence conserved between all betasatellites (known as the satellite conserved region; 1264 to 15) that contains a predicted stem-loop structure with the nonanucleotide sequence TAATATTAC forming part of the loop. The sequence showed between 77.1% and 89% nucleotide sequence identity to isolates of Tomato leaf curl Java betasatellite (ToLCJaB) available in the databases (4 sequences available at this time) with the highest identity 89.4% to an isolate originating from Nepal isolated from papaya [23]. A phylogenetic tree, based on an alignment of the complete nucleotide sequence of the betasatellite isolated from tomato with selected betasatellite sequences available in the databases is shown in Figure 2B. This shows J29.6 to segregate with isolates of ToLCJaB, confirming its identification as an isolate of ToLCJaB for which the isolate descriptor [Japan:Komae:2011] ([JR:Kom:11]) is proposed.

#### 2.1.3. Analysis of Alphasatellites

The two alphasatellite clones obtained, isolated from tomato infected with TYLCV (isolate J25.16; acc. no. KC677735) and tomato infected with AYVV and ToLCJaB (isolate J29.26; KC677736), were determined to be 1,359 and 1,357 nt, respectively, in length. The two sequences show 99% nucleotide sequence identity and the typical arrangement of alphasatellites, containing a single large open reading frame with a coding capacity of 315 amino acids in the virion-sense (coordinates 80-1027 for both), a region of sequence rich in adenine (coordinates 1043-1236) with 51% adenine content and a predicted hairpin structure containing the nonanucleotide sequence motif TAGTATTAC, within the loop, typical of nanoviruses [24]. The two sequences showed the highest levels of nucleotide sequence identity (94.1% to 98.1%) to isolates of Sida yellow vein China alphasatellite (SiYVCNA) available in the databases (3 available at this time) with the highest levels of identity to an isolate (KC282643) originating from Nepal [23]. To all other alphasatellite sequences the levels of identity were below 88%. This indicates that the alphasatellites identified here are isolates of SiYVCNA. A phylogenetic analysis, based upon an alignment of the complete nucleotide sequences of the alphasatellites identified here with all available isolates of SiYVCNA and selected other alphasatellite sequences is shown in Figure 2C. In the tree the alphasatellite isolates from Japan segregate with the previously characterized SiYCCNA isolates, confirming their identification as isolates of SiYCCNA.

### 2.2. Discussion

TYLCV is probably the most serious pathogen of tomato at this time, which has spread to most tomato growing regions across the warm/temperate parts of the world, including Japan [19]. The virus was first reported in Japan in 1998 [20] and is a serious constrain to tomato cultivation there [25,26]. Although AYVV is reported to cause disease of tomato in Indonesia [27], China (Guangxi province; AJ558120) and on the Okinawa Islands of Japan [23], the virus has not previously been reported on mainland Japan. In Indonesia and on Okinawa the virus is associated with Ageratum yellow vein betasatellite (AYVB) and, for Indonesian isolates, the combination AYVV with AYVB was shown experimentally to induce severe symptoms in tomato [27].

No alphasatellites have previously been identified in Japan despite the extensive recent efforts to identify both begomoviruses and betasatellites in the country [24,25,26]. The apparent absence of alphasatellites, despite the presence of betasatellites, was noted as far back as when the diversity of alphasatellites was first investigated [24]. The alphasatellite identified here, SiYCCNA, has previously only been reported in Asia (China and Nepal). In Nepal the alphasatellite was in association with AYVV and ToLCJaB [23], as is the case for one of the plants examined here, and in China the satellite was associated with *Kenaf* *leaf* *curl* *virus* (FN806777), *Malvastrum* *yellow* *vein* *Baoshan* *virus* (FN806779) and Malvastrum yellow vein Yunnan betasatellite (FN806780). TYLCV is not normally associated with either alpha- or betasatellites. However, a distinct strain of TYLCV originating from Oman has been shown to be associated with both a betasatellite (Tomato leaf curl betasatellite) and an unusual alphasatellite (Ageratum yellow vein Singapore alphasatellite) [21]. The low levels of sequence divergence between all isolates of SiYCCNA, as well as the lack of earlier reports of alphasatellites in the country, suggest that this alphasatellite has only recently spread into Japan.

Ueda *et* *al*. [28] have shown experimentally that a TYLCV isolate from mainland Japan and the betasatellite occurring in Okinawa (AYVB) can interact and induce enhanced symptoms in plants. Several other begomoviruses have been reported cause diseases of tomato in Japan, including HYVMV, TbLCJV and the associated betasatellite Honeysuckle yellow vein mosaic betasatellite [17,18]. The identification of yet another begomovirus and distinct betasatellite is thus a worrying development with the possibility of recombination and component exchange leading to viruses with greater pathogenicity that may cause more problems for agriculture. The precise contribution alphasatellites make in begomovirus/betasatellite infections remains unclear. Some alpasatellites have been shown to reduce the symptoms induced by virus infection by specifically down-regulating betasatellite levels, thus possibly allowing the infected plant to survive longer and allowing greater time for vector-mediated spread [21]. Another study has shown that at least some alphasatellites encode a Rep protein which can suppress post-transcriptional gene silencing, indicating that they are involved in overcoming plant host defenses [29]. It is therefore difficult to predict what effect the introduction of an alphasatellite into the agroecosystem of Japan will have.

## 3. Experimental Section

### 3.1. Sample Collection, Cloning and Sequencing

Leaves of four symptomatic tomato plants were collected from two distinct locations (A: Central Komae and B: South West of Komae) and genomic DNA was extracted using a PhytoPure Plant DNA Extration Kit (GE Amersham Biosciences, Little Chalfont, UK). Initial analysis by PCR with a pair of diagnostic primers [30] showed the presence of a begomovirus. A pair of primers for amplification of the full-length begomovirus genome (or DNA A component; (TYLCVF 5' GTTGAGCTCTTAGCTGCCTGAA 3' and TYLCVR 5' AAGAGCTCAACAGATGTCAAG3') was designed from the partial sequence obtained with the product of the diagnostic primers. These primers, together with BF/FR, beta01/beta02 and DNA101/DNA102 [31,32,33] were used to PCR amplify the full-length begomovirus, betasatellite and alphasatellite components, respectively, from all infected plant samples. Multiple clones were obtained, and one clone amplified with each primer pair (designated clones J25.6 and J25.16 for the begomovirus and alphasatellite, respectively, from tomato plant A and clones J29.22, J29.6 and J29.26 for the begomovirus, betasatellite and alphasatellite, respectively, from tomato plant B; Figure 1), were selected and sequenced entirety in both orientations (Macrogen, Tokyo, Japan).

### 3.2. Sequence Analysis

Sequences were assembled and analyzed with the aid of the Lasergene package of sequence analysis software (DNAStar Inc., Madison, WI, USA), and multiple sequence alignments were performed using Clustal X [34]. Phylogenetic trees were constructed using the Neigbour Joining algorithm of Clustal X and displayed, manipulated and printed using Treeview [35]. Sequences of begomoviruses and associated satellites used in the analyses were obtained from the EMBL nucleotide sequence database.

## 4. Conclusions

This is the first report of the occurrence of an alphasatellite in Japan, as well as the first report of AYVV occurring on the mainland. The results indicate that the alphasatellite introduction has occurred relatively recently. However, what effect the presence of an alphasatellite may have on agriculture in Japan is unclear. Both TYLCV and AYVV are known pathogens of tomato and other agriculturally important plants species. The increasing diversity of begomoviruses and their associated satellites in Japan is of concern due to the possibility of more virulent viruses or virus-satellite combinations evolving that may increase losses to tomato production and come to affect other crops. Greater efforts need to be made by the plant protection services to prevent any further incursions. 
